# Color Blindness

**Published:** 1860-02

**Authors:** 


*Color Blindness.*—At a recent meeting of the *British Association for the Advancement of Science*, held at Aberdeen, Professor George Wilson, M. D., of Edinburgh, read a paper entitled, "Some Statistics on Color Blindness." This term, he said, was not to be applied to a disease, but a remarkable type of vision. In giving an explanation of the nature of this visual peculiarity, he narrated some remarkable instances. "Color-blind people," he said, "do not see the red in pink—they think it is white; and if we darken red with black, they stop seeing any red in it and call it black before we do." This peculiarity is chiefly shown in the confounding of red and green, and in the matching or confounding of dark red and brown, in confounding red and black, and different shades of the same color. To illustrate this peculiarity, the Professor introduced the case of an upholsterer in Edinburgh, who covered a coffin with scarlet cloth; and of agentleman of the same city, who asked a lady with a red velvet bonnet, for whom she was in mourning. The earliest recorded case of the sort is mentioned in an old number of the *Philosophical Transactions.* In the days when etiquette forbade any one but a clergyman, physician, or lawyer, to appear in a black coat,—at a wedding, the bride's father was about to dismiss the bridegroom for appearing in this dress. He was only saved by the remonstrances of the fair bride, who appealed to the guests to satisfy her father that her lover had on a wedding raiment of the height of fashion,—claret-colored. Many people exhibit this peculiarity in mistaking the color of flowers. Dalton, the chemist, discovered that he was color-blind by hearing a geranium, which he took to be blue, called red by various persons. Dugald Stewart, the philosopher, could only distinguish a cherry from its leaves by its form. We once knew a man who brought a black ribbon home to his wife instead of a scarlet one.—*Nashville Journal of Medicine and Surgery.*

